# Targeting metabolic inflammation in type 2 diabetes mellitus through exercise: multi-level mechanisms of insulin resistance reversal and precision clinical translation — a review

**DOI:** 10.3389/fimmu.2026.1886943

**Published:** 2026-08-03

**Authors:** Li Wu

**Affiliations:** College of Sports Science, Jishou University, Jishou, China

**Keywords:** exercise, insulin resistance, metabolic inflammation, NLRP3 inflammasome, type 2 diabetes mellitus

## Abstract

Insulin resistance (IR) remains the central pathophysiological driver of type 2 diabetes mellitus (T2DM) and is inadequately targeted by current pharmacotherapies. Emerging evidence in immunometabolism identifies metabolic inflammation (meta-inflammation) as a critical mechanistic bridge between chronic nutrient excess and IR progression, primarily through IKKβ/NF-κB and JNK signaling cascades, NLRP3 inflammasome activation, mitochondrial dysfunction, and lipotoxicity. Exercise intervention counteracts these processes via coordinated multi-tiered mechanisms, including AMPK-mediated insulin sensitization, myokine-driven anti-inflammatory modulation, mitophagy-based mitochondrial quality control, liver–muscle–adipose metabolic crosstalk, and epigenetic reprogramming of inflammatory transcriptional responses. However, clinically significant interindividual variability in exercise responsiveness—termed exercise non-response—limits the effectiveness of population-averaged prescription guidelines. This heterogeneity is attributable to genetic polymorphisms, baseline inflammatory burden, gut microbiota composition, and suboptimal exercise protocol design. This review synthesizes mechanistic evidence linking meta-inflammation, exercise, and IR, and proposes a precision exercise medicine framework integrating multi-omics phenotyping (genomics, metabolomics, and microbiome profiling) with data-driven clinical decision-support tools. This approach aims to translate mechanistic insights into individualized therapeutic strategies for the long-term management of T2DM.

## 1 Introduction

Type 2 diabetes mellitus (T2DM) has become one of the major global challenges in chronic disease prevention and management. According to the International Diabetes Federation (IDF), more than 500 million adults worldwide are currently living with T2DM, and this number is projected to increase substantially over the next two decades with continued population aging, accelerated urbanization, and the widespread prevalence of sedentary lifestyles (1). The microvascular and macrovascular complications associated with T2DM not only markedly impair patients’ quality of life, but also impose a substantial economic burden on healthcare systems worldwide because of the long-term and intensive demands of disease management (2). Although modern glucose-lowering therapies, including metformin, sodium-glucose cotransporter 2 (SGLT2) inhibitors, and glucagon-like peptide-1 (GLP-1) receptor agonists, are effective in improving glycated hemoglobin (HbA1c), they primarily target glycemic manifestations and remain limited in fundamentally reversing insulin resistance (IR), the central pathophysiological basis of T2DM (3). Therefore, elucidating the mechanisms underlying IR and developing interventions targeting its upstream determinants remain urgent priorities in metabolic disease research.

**Figure 1:**
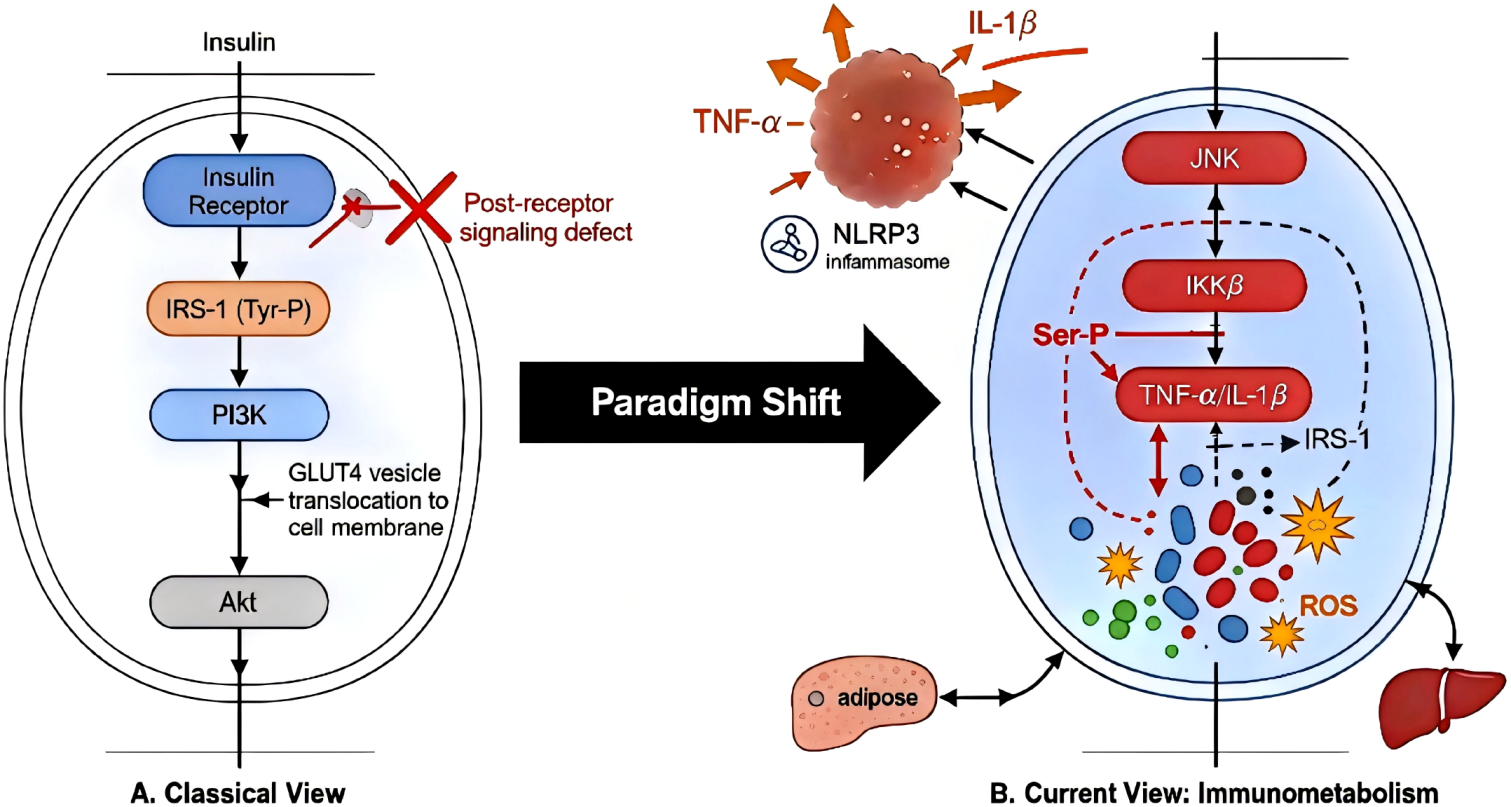
Paradigm shift in insulin resistance research: from single post-receptor signaling defects to meta-inflammation-mediated multi-organ immunometabolic Dysregulation.

Previous studies have primarily attributed IR to impaired phosphorylation cascades at the post-receptor level of the insulin signaling pathway, specifically defective PI3K/Akt axis transduction and the consequent impairment of glucose transporter type 4 (GLUT4) translocation (4). Over the past two decades, with the emergence of immunometabolism as a discipline, the research paradigm in this field has undergone a profound transformation. Metabolic inflammation (meta-inflammation) has been increasingly recognized as a central pathophysiological mechanism driving both the onset and progressive deterioration of IR (5). This conceptual advance has deepened rather than supplanted the understanding of classical PI3K/Akt signaling impairment, expanding the pathological landscape of IR from isolated intracellular signaling defects to a cross-tissue dysregulation of immune–metabolic interactions (**Figure 1**). Meta-inflammation is essentially a chronic low-grade, systemic inflammatory state induced by nutrient excess and obesity, characterized by the persistent activation of immune cells, particularly macrophages, in adipose tissue (especially visceral fat), the liver, and skeletal muscle, along with the aberrant release of pro-inflammatory cytokines including TNF-α, IL-6, and IL-1β (6). This inflammatory state is not a passive consequence of metabolic dysregulation; rather, it actively interferes with tyrosine phosphorylation of insulin receptor substrates (IRS-1/2) through IKKβ/NF-κB and JNK signaling cascades, while concurrently inducing lipotoxicity and mitochondrial oxidative stress, ultimately establishing a pathological positive feedback loop in which inflammation exacerbates IR, IR promotes hyperglycemia and lipotoxicity, and the resulting metabolic stress further amplifies inflammation (7). Notably, the NLRP3 inflammasome, functioning as a critical amplification node within this cycle, mechanistically couples metabolic signals to immune activation through the proteolytic maturation and secretion of IL-1β, and has consequently emerged as an important therapeutic target for IR intervention in recent years.

Regular physical exercise, as a cornerstone non-pharmacological intervention in T2DM management, encompasses physiological dimensions that extend well beyond the conventional framework of energy metabolism (8). As a periodically controllable physiological stressor, exercise reshapes immune–metabolic homeostasis through multi-tiered molecular networks: the activation of myokine networks induced by skeletal muscle contraction, the adaptive remodeling of mitochondrial biogenesis and fusion–fission dynamics, and the systemic modulation of gut microbiota composition and function collectively constitute the core mechanistic pathways through which exercise dismantles metabolic inflammation and restores insulin signal transduction (9, 10). Nevertheless, a substantial gap persists between the clinical consensus that “exercise is beneficial” and its precision clinical application, manifested across three key dimensions. First, the dose–response relationship governing the anti-inflammatory and insulin-sensitizing effects of exercise — encompassing the optimization of exercise modality, intensity, frequency, and duration — still lacks consistent evidence from high-quality randomized controlled trials. Second, the critical molecular targets mediating exercise-induced anti-inflammatory effects and the cross-tissue signal transduction mechanisms underlying their interactions remain to be systematically elucidated. Third, a considerable proportion of individuals with T2DM exhibit attenuated responses to standard exercise interventions — a phenomenon termed “exercise resistance” (defined as a metabolic improvement significantly below the population mean despite adherence to a standardized exercise protocol) — whose genetic, epigenetic, and microbiome-level determinants have not been adequately characterized, thereby substantially impeding the development and clinical application of biomarker-guided individualized exercise prescriptions.

It is worth noting that previous reviews of this topic have predominantly focused on individual mechanistic dimensions - some concentrating on myokine networks (11), others emphasizing the mitochondria-AMPK axis (12), and still others centering on the gut microbiota–gut–brain axis (13), with few attempts to employ metabolic inflammation as a unifying analytical framework that systematically integrates these multi-tiered mechanisms and rigorously bridges them to clinical translation pathways. The present review seeks to address this gap at the level of conceptual integration.

Against this backdrop, the present review aims to address this gap at the level of conceptual integration. While it is well established that exercise improves insulin sensitivity through individual pathways such as the AMPK–mitochondrial axis or myokine signaling, these mechanisms have typically been discussed in isolation. In contrast, we first propose a ‘muscle–immune–metabolic axis’ integrative model centered on meta-inflammation, which seeks to reconsolidate these disparate molecular mechanisms into a unified analytical framework. Building upon this foundation, we further develop a ‘precision exercise medicine’ framework that integrates multi-omics data with real-time physiological monitoring to address the clinical heterogeneity in T2DM patients’ responses to exercise interventions. This paradigm shift from a ‘one-size-fits-all’ approach toward a dynamic, data-driven system not only represents a fundamental advance over the existing literature but also provides a concrete roadmap for the future clinical translation of individualized exercise prescriptions.

Against this background, this review aims to delineate, with metabolic inflammation as the central axis, the dynamic interplay between exercise intervention and insulin resistance in T2DM, and to reconstruct a multi-tiered mechanistic map of this metabolic–immune interaction network. Specifically, this review will: (1) dissect the molecular mechanisms through which metabolic inflammation directly disrupts insulin signal transduction via IKKβ/NF-κB and JNK kinase cascades and NLRP3 inflammasome activation, and elucidate their synergistic interplay with mitochondrial dysfunction and lipotoxicity; (2) drawing on advances in myokinome research and mitochondrial dynamics, systematically delineate the multi-tiered molecular mechanisms through which exercise remodels immune–metabolic homeostasis and enhances insulin sensitivity; (3) critically appraise the available randomized controlled trial evidence for clinical exercise interventions, and systematically evaluate the differential efficacy and practical limitations of distinct exercise modalities, including aerobic exercise, resistance training, and high-intensity interval training (HIIT); and (4) outline prospective directions for precision exercise medicine driven by multi-omics integration — encompassing genomics, epigenomics, and microbiomics — thereby providing a theoretical framework and academic reference for the development of biomarker-stratified individualized exercise prescription systems and the identification of novel therapeutic targets in metabolic disease.

## 2 Meta-inflammation and insulin resistance in type 2 diabetes mellitus

The pathological progression of T2DM is not merely a disorder of glucose metabolism, but rather a systemic immune remodeling driven by nutrient excess that encompasses the full spectrum of metabolic tissues (14). Meta-inflammation, serving as the central nexus linking energy surplus to insulin resistance (IR), is pathologically defined by a series of maladaptive molecular cascades initiated within the innate immune system under conditions of sustained lipotoxicity and oxidative stress (15). In contrast to the self-limiting nature of conventional immune responses, this inflammatory state persists in chronic low-grade activation across metabolic tissues, exerting a progressive erosive effect on the insulin signaling network (16). This section systematically dissects the multi-tiered mechanisms underlying the pathological coupling of meta-inflammation and IR across three dimensions: immune cell polarization and tissue microenvironment remodeling; stress kinase-mediated disruption of molecular signaling; and NLRP3 inflammasome-driven lipotoxic signal transduction.

### 2.1 Pathological remodeling of immune cell polarization and tissue microenvironment

Adipose tissue plays a pivotal “sentinel” role in the initiation of meta-inflammation. Under conditions of obesity, sustained lipid accumulation drives marked adipocyte hypertrophy, with cell diameters exceeding the effective oxygen diffusion radius of adjacent capillaries, thereby inducing local tissue hypoxia and abnormal accumulation of extracellular matrix mechanical tension (17). The hypoxic microenvironment activates hypoxia-inducible factor-1α (HIF-1α), which upregulates the expression of chemokines including monocyte chemoattractant protein-1 (MCP-1, also known as CCL2), driving the robust recruitment of circulating monocytes into adipose tissue and their subsequent *in situ* differentiation into adipose tissue macrophages (ATMs) (18). Critically, the central feature of this recruitment process lies not merely in the numerical expansion of ATMs, but in the systematic shift of their functional phenotype. Within the lipotoxic microenvironment, free fatty acids (FFAs), oxidized low-density lipoprotein (oxLDL), and tumor necrosis factor-α (TNF-α) act in concert to drive the phenotypic transition of ATMs from an anti-inflammatory, tissue-reparative state (canonically defined as the M2 phenotype) toward a pro-inflammatory state (canonically defined as the M1 phenotype) (19, 20). It should be noted that the classical M1/M2 binary classification framework, though a considerable simplification — given that the underlying molecular heterogeneity is far richer than this dichotomy can capture — nonetheless remains a practical analytical tool for characterizing the polarization state of adipose tissue inflammation (21). M1-polarized ATMs release TNF-α, interleukin-6 (IL-6), and interleukin-1β (IL-1β) in a paracrine manner, not only altering the endocrine function of adipose tissue itself — manifested as the suppression of protective adiponectin secretion and the aberrant upregulation of resistin — but also amplifying and sustaining the output of pro-inflammatory signals to metabolic tissues throughout the body (22).

These inflammatory effects are characterized by marked tissue heterogeneity and hierarchical propagation. Pro-inflammatory cytokines generated by visceral adipose tissue act directly on the liver via the portal circulation, activating tumor necrosis factor receptors (TNFR) in hepatocytes while concurrently inducing endoplasmic reticulum (ER) stress and mitochondrial dysfunction, jointly resulting in the selective impairment of hepatic insulin signaling — a condition referred to as hepatic insulin resistance (23). This impairment is functionally characterized by the inability of insulin to effectively suppress hepatic gluconeogenesis, while its lipogenic signaling pathway is paradoxically preserved or even enhanced, a phenomenon termed paradoxical insulin signaling. Concurrently, skeletal muscle — the principal site of glucose disposal in the body, accounting for approximately 75% of postprandial glucose uptake — is subjected to the combined insult of circulating pro-inflammatory cytokines and locally accumulated lipid intermediates, specifically ceramide and diacylglycerol (DAG) (24). This mechanism, whereby distal inflammatory signals synergize with local lipotoxicity, progressively amplifies the localized immune activation of adipose tissue into a systemic insulin resistance phenotype, establishing a critical pathological foundation for the global metabolic dysregulation observed in T2DM (25). Nevertheless, inflammatory activation at the tissue level must ultimately be transduced through intracellular molecular events before it can exert substantive interference with insulin signaling. This pathological state driven by immune cell polarization constitutes the direct target through which exercise interventions exert their restorative effects via the ‘muscle–immune–metabolic axis,’ as elaborated in the subsequent sections.

### 2.2 Molecular interference mechanisms of kinase signaling cascades and structural impairment of insulin signaling

The transduction of tissue-level inflammatory activation into intracellular molecular events converges critically on the activation of two core stress kinase axes. c-Jun N-terminal kinase (JNK) and inhibitor of nuclear factor-κB kinase β (IKKβ) serve as the central molecular decoders through which inflammatory signals penetrate the insulin signaling network, playing indispensable roles in meta-inflammation-induced IR (26, 27).

The pro-inflammatory cytokines TNF-α and IL-1β engage their respective transmembrane receptors to activate adaptor proteins including TNF receptor-associated factor 6 (TRAF-6) and receptor-interacting protein kinase 1 (RIP1), which in turn trigger the phosphorylation and activation of JNK and IKKβ, respectively (28). The critical shared substrate of both kinases is insulin receptor substrate-1 (IRS-1): JNK primarily catalyzes phosphorylation at serine 307 of IRS-1 (Ser307; corresponding to Ser312 in humans), while IKKβ exerts analogous effects through phosphorylation of multiple serine residues on IRS-1. This post-translational modification impairs the integrity of insulin signaling through multiple mechanisms (29). First, phosphorylated IRS-1 undergoes conformational changes that significantly reduce the substrate presentation efficiency of its phosphotyrosine-binding (PTB) domain toward insulin receptor kinase (IRK), thereby suppressing the activation of the phosphoinositide 3-kinase/protein kinase B (PI3K/Akt) pathway. Second, serine-phosphorylated IRS-1 is preferentially recognized by F-box-containing E3 ubiquitin ligases, such as Fbw8, and subsequently degraded via the ubiquitin–proteasome pathway, further diminishing insulin signaling capacity at the level of protein abundance (30). In addition, under inflammatory conditions, the expression of protein tyrosine phosphatase 1B (PTP1B) is markedly upregulated; by catalyzing the dephosphorylation of phosphotyrosine residues on IRS-1, PTP1B synergistically amplifies the attenuation of insulin signaling in concert with the aforementioned JNK/IKKβ-mediated mechanisms. Notably, PTP1B itself has emerged as an important therapeutic target in T2DM drug development (31).

From a pathophysiological perspective, this inflammation-mediated signal blockade is characterized by two critically important features. The first is reversibility: unlike permanent signaling defects caused by genetic mutations or structural receptor damage, the serine phosphorylation state of IRS-1 is subject to dynamic regulation by the balance between kinases and phosphatases, such that a reduction in upstream inflammatory signals theoretically confers the potential for functional restoration of the pathway (32). The second is dynamism: the extent and duration of this signal blockade are highly dependent on the maintenance of the pro-inflammatory microenvironment and are exquisitely sensitive to changes in cellular metabolic status, such as exercise-induced activation of AMP-activated protein kinase (AMPK) (33). Together, these two properties reveal that, by eliminating upstream inflammatory drivers or activating antagonistic metabolic pathways, it is possible to substantially reshape cellular insulin sensitivity without altering the genomic background. Nevertheless, an understanding of inflammation-driven IR solely at the kinase level remains incomplete. At a level upstream of cellular sensing of metabolic danger signals, NLRP3 inflammasome activation provides the core amplification mechanism that converts lipid metabolic dysregulation into sustained inflammatory output.

### 2.3 The NLRP3 inflammasome and insulin resistance in type 2 diabetes mellitus

If the JNK/IKKβ axis represents the core executive machinery through which inflammation disrupts insulin signaling, the NLRP3 inflammasome constitutes the intracellular sensing platform that autonomously amplifies metabolic danger signals — damage-associated molecular patterns (DAMPs) — into sustained inflammatory output. Its activation follows a well-established two-signal model. During the priming phase, free fatty acids (FFAs) and their metabolic derivatives, generated under high-fat dietary conditions, activate Toll-like receptor 4 (TLR4), triggering transcriptional activation of the nuclear factor-κB (NF-κB) pathway and upregulating basal expression levels of NLRP3 protein itself, pro-IL-1β, and pro-IL-18, thereby establishing the molecular prerequisites for subsequent inflammasome assembly. Following this transcriptional priming, ceramide accumulated through persistent lipid metabolic dysregulation and excess mitochondrial reactive oxygen species (mtROS) engage two intersecting pathways to trigger the recruitment and oligomerization of the NLRP3 complex, initiating the activation phase. Ceramide promotes lysosomal membrane permeabilization (LMP), leading to the aberrant release of cathepsin B into the cytosol; mtROS directly drive NLRP3 oligomerization and the subsequent recruitment of apoptosis-associated speck-like protein containing a CARD (ASC) through oxidative modification of cysteine residues on NLRP3 (34). These two converging pathways synergistically trigger the autocatalytic activation of caspase-1, which cleaves pro-IL-1β and pro-IL-18 into their mature forms; the processed cytokines are then released extracellularly through membrane pores formed by gasdermin D (GSDMD), further amplifying tissue inflammation and reinforcing the pathological positive feedback driving IR (35). Within this NLRP3-mediated inflammatory activation cycle, mitochondria do not function merely as generators of reactive oxygen species, but simultaneously serve dual roles as metabolic state sensors and inflammatory signal amplifiers. Under sustained lipotoxic stress, mitochondria undergo pronounced morphofunctional remodeling: the suppression of pro-fusion proteins (mitofusin-1/2, MFN1/2) and the hyperactivation of the pro-fission GTPase dynamin-related protein 1 (DRP1) create a dynamic imbalance that drives progressive fragmentation of the mitochondrial network. Fragmented mitochondria exhibit reduced coupling efficiency of the electron transport chain (ETC), further exacerbating mtROS leakage and thereby establishing a subcellular positive feedback loop in which mtROS accumulation sustains NLRP3 activation, driving abundant IL-1β release that maintains the pro-inflammatory microenvironment and further amplifies mtROS generation (36), enabling meta-inflammation to become self-perpetuating even after the attenuation of the initial stimulus.

Mitochondrial structural and functional disruption ultimately results in substantive impairment of metabolic flexibility — defined as the capacity of cells to efficiently switch between lipid oxidation and glucose oxidation in response to substrate availability and energy demand. Under conditions of metabolic health, this capacity is dynamically maintained through AMPK activation and peroxisome proliferator-activated receptor γ coactivator-1α (PGC-1α)-mediated mitochondrial biogenesis (37). However, in the context of T2DM with concurrent meta-inflammation, the dynamic imbalance of mitochondrial fusion–fission leads to excessive mitochondrial fragmentation, accompanied by sustained reduction in mitochondrial membrane potential (ΔΨm), diminished oxidative phosphorylation efficiency, and impaired fatty acid β-oxidation capacity. These resting-state mitochondrial defects prevent efficient upregulation of oxidative phosphorylation flux during the transition from rest to exercise stress in skeletal muscle cells, resulting in marked metabolic inflexibility. This metabolic inflexibility is not merely a functional manifestation of impaired skeletal muscle glucose uptake, but represents a critical molecular substrate underlying the attenuated adaptive metabolic response to exercise-induced stimuli - driven by the mutually reinforcing pathological network comprising NLRP3 inflammasome activation, mitochondrial network fragmentation, and oxidative stress (38). At this juncture, three interconnected pathological tiers — immune cell phenotypic polarization and adipose tissue microenvironment remodeling; structural impairment of insulin signaling co-mediated by the JNK/IKKβ/PTP1B triad; and the coupling of NLRP3 inflammasome activation with mitochondrial metabolic flexibility exhaustion — collectively constitute the multi-tiered pathological network through which meta-inflammation undermines insulin sensitivity. Importantly, each of these three mechanistic layers harbors theoretical interventional accessibility: the functional tunability of M1/M2 polarization, the dynamic reversibility of IRS-1 serine phosphorylation, and the inducible plasticity of mitochondrial biogenesis together provide multi-tiered molecular targets for physiological interventions centered on exercise — a framework that will be systematically elaborated in subsequent sections.

In summary, the pathological landscape of insulin resistance in T2DM is not a simple superposition of individual molecular events, but rather a pathological positive-feedback network constituted by an ‘immune–metabolic–organellar’ triad. Specifically, M1 macrophage polarization and the lipotoxic microenvironment within adipose tissue exert structural interference on the insulin signaling pathway through the IKKβ/JNK kinase axis. Concurrently, the imbalance in mitochondrial dynamics (excessive fission) and NLRP3 inflammasome activation form a subcellular cascade of ‘mtROS generation–inflammatory amplification–further mitochondrial damage,’ which not only sustains the systemic low-grade inflammatory state but also ‘locks’ IRS-1 into functional inactivation through persistent serine phosphorylation.

On this basis, we conceptualize exercise intervention as a ‘multi-node coordinated restoration’ of this pathological network. Rather than merely serving as a means of energy expenditure, exercise reverses the aforementioned pathological network across three dimensions through activation of the AMPK/SIRT1/PGC-1α axis. First, the anti-inflammatory effects of myokines (e.g., IL-6) block pro-inflammatory signaling interference with insulin receptor substrates at its source. Second, AMPK-driven mitophagy and mitochondrial biogenesis restore mitochondrial network integrity, fundamentally disrupting the activation threshold of the NLRP3 inflammasome. Third, cross-organ metabolic crosstalk enhances the clearance of lipid intermediates (e.g., ceramides, DAG), thereby relieving the negative allosteric regulation imposed on the insulin signaling pathway. This transformation from a ‘pathological positive-feedback loop’ to a ‘physiological restorative circuit’ constitutes the systems-biology rationale underlying exercise-mediated improvement of insulin resistance in T2DM.

## 3 Exercise attenuates meta-inflammation and improves insulin signal transduction in T2DM through multi-pathway synergistic mechanisms

The beneficial effects of exercise on T2DM extend far beyond caloric expenditure; they represent a complex feedback network in which skeletal muscle orchestrates the multi-pathway, synergistic repair of damaged nodes within the insulin signaling cascade. Through the coordinated engagement of direct metabolic sensitization, myokine-mediated indirect anti-inflammatory effects, cross-organ metabolic crosstalk, and long-term epigenetic remodeling, exercise reverses the sustained suppression of insulin signal transduction imposed by meta-inflammation at multiple molecular nodes.

### 3.1 Direct metabolic sensitization: AMPK-driven insulin-independent glucose uptake and lipotoxic substrate clearance

AMP-activated protein kinase (AMPK) is the central kinase driving the direct metabolic sensitization induced by exercise. Exercise triggers a rapid increase in the intramuscular AMP/ATP ratio, and the resulting AMPK activation promotes the translocation of glucose transporter type 4 (GLUT4)-containing storage vesicles to the plasma membrane in a manner independent of insulin receptor signaling, through phosphorylation of Tre-2/BUB2/CDC16 domain family member 1 (TBC1D1) and its homolog TBC1D4 (also known as AS160), thereby inhibiting their GTPase-activating protein (GAP) activity toward Rab GTPases and enabling immediate insulin-independent glucose uptake. It should be noted that TBC1D1 plays a particularly prominent role in skeletal muscle, whereas TBC1D4 is of greater importance in adipocytes and cardiomyocytes (39). Furthermore, exercise-induced intracellular calcium transients activate Ca²^+^/calmodulin-dependent protein kinase II (CaMKII), which synergistically promotes GLUT4 translocation through an AMPK-independent bypass mechanism; together, these two pathways constitute a dual-axis drive for exercise-induced glucose uptake in skeletal muscle (40). This bypass mechanism carries particular compensatory significance in patients with T2DM in whom canonical insulin signaling is impaired.

Concurrently, AMPK phosphorylates acetyl-CoA carboxylase (ACC), relieving its allosteric inhibition of carnitine palmitoyltransferase 1 (CPT1) and thereby accelerating the mitochondrial import and β-oxidation of long-chain fatty acids. This process effectively reduces the intramuscular accumulation of lipotoxic intermediates, including ceramide and diacylglycerol (DAG), which in turn relieves the negative allosteric regulation exerted by these substrates on IRS-1 at the level of molecular conformation, providing the necessary molecular basis for the restoration of normal tyrosine phosphorylation of insulin receptor substrates (41). Therefore, AMPK-mediated clearance of lipotoxic intermediates represents not merely a metabolic improvement but, by relieving the negative allosteric regulation imposed on IRS-1, provides the requisite molecular and structural foundation for the subsequent exertion of anti-inflammatory effects—exemplifying the synergy between metabolic and immune restoration. However, if the activity of pro-inflammatory stress kinases IKKβ and JNK persists, the direct metabolic sensitization described above will be substantially undermined by continued serine phosphorylation of IRS-1, indicating that repair of metabolic pathways alone is insufficient to achieve durable insulin sensitization and must be complemented by the concurrent engagement of upstream anti-inflammatory effects.

### 3.2 Indirect anti-inflammatory effects: synergistic remodeling of the myokine network and mitochondrial quality control

Notably, the immune system is not a passive recipient of exercise intervention but rather an active regulator of metabolic homeostasis. Through the myokine network and mitochondrial quality control, exercise directly reshapes the phenotypic polarization of immune cells. This ‘muscle–immune–metabolic’ crosstalk enables the immune system to sense the metabolic stress induced by exercise and shift from a pro-inflammatory M1 phenotype toward an anti-inflammatory M2 phenotype, thereby achieving coordinated immune and metabolic restoration at the molecular level. Accordingly, the anti-inflammatory effects of exercise are principally mediated through the ‘Muscle–Immune–Metabolic Axis,’ in which the myokine network and the mitochondrial quality control system constitute the two core pathways of this axis.

At the level of myokines, exercise-induced skeletal muscle-derived IL-6 represents the anti-inflammatory myokine with the most robust supporting evidence to date. It is important to emphasize that exercise-induced IL-6 is fundamentally distinct in its functional properties from IL-6 derived from adipose tissue and immune cells: the former is abundantly secreted by skeletal muscle during exercise and acts primarily through classical receptor signaling to stimulate hepatic production of IL-1 receptor antagonist (IL-1Ra) and the anti-inflammatory cytokine IL-10, while also promoting the secretion of glucagon-like peptide-1 (GLP-1), thereby systemically reducing circulating levels of TNF-α and IL-1β and directly antagonizing the downstream effector molecules of the NLRP3 inflammasome (10). Furthermore, exercise-induced systemic anti-inflammatory signals (e.g., IL-1Ra and IL-10) are not merely anti-inflammatory factors but also serve as critical ‘metabolic sensors’ that, by antagonizing NLRP3 inflammasome activation, directly block the conversion of metabolic danger signals (DAMPs) into sustained inflammatory output. This direct coupling of immune responses to metabolic improvement enables the immune system to sense exercise-induced metabolic stress in real time and translate it into protective signals that suppress inflammation and restore insulin sensitivity, thereby playing a central regulatory role within the ‘Muscle–Immune–Metabolic Axis.’ In addition, irisin has emerged as another candidate myokine of interest, demonstrating, in animal models, the functional potential to suppress NF-κB signaling pathway activation and promote adipose tissue browning (42); however, its physiological significance and effective circulating concentrations in humans remain subjects of substantial debate, and interpretation of the available evidence warrants considerable caution.

At the level of mitochondrial quality control, exercise-activated AMPK and sirtuin 1 (SIRT1) act in sequential concert to co-activate PGC-1α. AMPK initiates the transcriptional activity of PGC-1α through direct phosphorylation at Thr177 and Ser538, while SIRT1 further augments its functional activity through deacetylation of lysine residues on PGC-1α, together establishing a dual-layer activation mode comprising phosphorylation and deacetylation. PGC-1α activation not only drives mitochondrial biogenesis but also promotes the selective autophagy of depolarized mitochondria (mitophagy) through upregulation of PTEN-induced putative kinase 1 (PINK1) and the E3 ubiquitin ligase Parkin (43, 44). This mitochondrial quality control process directly addresses the pathological mechanism of NLRP3 inflammasome activation described in Section 1.3. By clearing damaged mitochondria, it reduces the inflammatory signal ‘amplifier’ at its source, thereby achieving a cross-level integration from organellar functional repair to systemic anti-inflammatory effects. This quality control process carries critical anti-inflammatory significance: dysfunctional mitochondria continuously generate excess mtROS and release mitochondrial DNA (mtDNA), which, acting as endogenous DAMPs, can activate the cyclic GMP-AMP synthase–stimulator of interferon genes (cGAS–STING) pathway and the NLRP3 inflammasome. By enabling the timely elimination of such damaged mitochondria, PINK1/Parkin-mediated mitophagy interrupts the continuous release of mtROS and mtDNA at the source, thereby disrupting the subcellular positive feedback loop in which mtROS-driven NLRP3 inflammasome activation promotes IL-1β secretion and further amplifies mtROS generation, and fundamentally dismantling the core self-perpetuating mechanism of meta-inflammation.

### 3.3 Cross-organ interactions: systemic integration of multi-tissue metabolic remodeling

The metabolic sensitizing effects of exercise are not confined to skeletal muscle; rather, they mediate systemic interactions among multiple organs through circulating myokines, hormones, and metabolic substrates, collectively forming a multidirectional feedback network encompassing skeletal muscle, the liver, adipose tissue, and the pancreatic islets.

In the liver, exercise activates hepatic AMPK, which suppresses the transcriptional expression of phosphoenolpyruvate carboxykinase (PEPCK) and glucose-6-phosphatase (G6Pase) through phosphorylation and inactivation of the transcription factor forkhead box protein O1 (FOXO1), while concurrently downregulating sterol regulatory element-binding protein-1c (SREBP-1c)-mediated *de novo* lipogenesis and promoting post-exercise hepatic glycogen resynthesis, thereby attenuating hepatic insulin resistance and steatosis through multiple complementary mechanisms (45).

In adipose tissue, regular exercise reduces total visceral fat mass and globally attenuates the pro-inflammatory polarization of ATMs, while simultaneously upregulating adiponectin secretion from adipocytes. Adiponectin activates AMPK in peripheral tissues primarily through AdipoR1 receptors — which are highly expressed in skeletal muscle — further reinforcing insulin-sensitizing effects, and activates peroxisome proliferator-activated receptor α (PPARα)-mediated fatty acid oxidation through AdipoR2 receptors — which are highly expressed in the liver — while concurrently suppressing NF-κB-driven inflammatory gene expression, thereby establishing a positive metabolic feedback loop among adipose tissue, skeletal muscle, and the liver (46).

In the pancreas, exercise-derived IL-6 promotes GLP-1 secretion from intestinal L cells; GLP-1 subsequently acts on GLP-1 receptors on pancreatic β cells to enhance glucose-stimulated insulin secretion (GSIS) (47). Concurrently, exercise-mediated attenuation of NLRP3 inflammasome activity directly alleviates the cytotoxic injury inflicted by IL-1β on β cells, providing structural protection at the level of inflammatory resolution for the partial restoration of islet function. Together with the metabolic improvements achieved at the skeletal muscle and hepatic levels, these pancreatic effects constitute a systemic reconstitution of insulin sensitivity.

### 3.4 Acute adaptation and long-term remodeling: exercise dose and epigenetic effects

The metabolic–immune repair effects induced by exercise exhibit marked temporal dependence, and their molecular basis can be understood across two distinct dimensions: acute adaptation and chronic remodeling. A single bout of acute exercise activates AMPK within hours, inducing transient GLUT4 translocation and myokine secretion, thereby achieving short-term improvements in glycemic control (48); however, the associated reductions in inflammatory signaling and restoration of IRS-1 function are both transient, gradually dissipating as the post-exercise recovery period extends.

In contrast, long-term regular exercise confers sustained benefits through multi-level adaptive remodeling. At the organelle level, sustained PGC-1α activation significantly increases mitochondrial density and oxidative enzyme activity in skeletal muscle under systematic aerobic training programs, while restoring the structural integrity of the mitochondrial network and the dynamic balance of fusion–fission cycling, thereby reducing basal mtROS generation at the structural level (49). At the epigenetic level, long-term exercise induces elevated DNA methylation at CpG sites within the promoter regions of inflammation-related genes in skeletal muscle — including TNF-α, IL-6, and NF-κB target genes — while concurrently mediating downregulation of histone deacetylase (HDAC) activity and increased chromatin accessibility at metabolic gene loci, thereby persistently elevating the organism’s transcriptional resistance threshold to inflammatory signals (50). Of clinical relevance, these epigenetic adaptations are partially reversible: following the cessation of long-term exercise, a proportion of these benefits progressively diminish through the process of detraining, underscoring that the continuity of exercise intervention is critical for sustaining long-term metabolic gains. This multi-level adaptive remodeling — spanning from organelle architecture to the epigenome — enables skeletal muscle to maintain a higher degree of metabolic flexibility in the face of intermittent nutritional excess, thereby achieving, at the systemic level, a long-term reconstitution from inflammation-driven metabolic dysregulation toward an insulin-sensitive homeostatic state.

In summary, through the deep integration of direct metabolic sensitization, myokine network-mediated indirect anti-inflammatory effects, multi-organ metabolic crosstalk, and epigenetic remodeling, exercise achieves targeted repair of the multi-node damage inflicted by meta-inflammation on the insulin signaling pathway, while realizing global reconstitution of the metabolic system via cross-organ interactions. The essence of exercise intervention lies in the following: through AMPK activation and myokine release, it simultaneously targets the ‘kinase interference axis (IKKβ/JNK)’ and the ‘inflammatory amplification axis (NLRP3/mtROS),’ thereby remodeling the pathological positive-feedback loop of ‘inflammation–insulin resistance–hyperglycemia’ into a physiological homeostatic cycle of ‘metabolic sensitization–anti -inflammation–insulin sensitivity restoration.’ Nevertheless, the realization of these effects is highly contingent upon the type, intensity, and duration of the exercise intervention, and interindividual responses to exercise exhibit substantial biological heterogeneity — factors that collectively underscore the necessity of precision exercise prescription, which will be systematically addressed in the following section.

## 4 Clinical heterogeneity and exercise resistance: from molecular response variability to the challenges of precision intervention

Although the anti-inflammatory and insulin-sensitizing mechanisms through which exercise functions as a molecular modulator are theoretically universal, clinical practice reveals that the metabolic responses of individuals with T2DM to exercise intervention exhibit substantial interindividual heterogeneity. A subset of patients demonstrates minimal improvement in insulin sensitivity following standardized exercise prescriptions, with some showing no statistically significant changes in metabolic indices — a phenomenon clinically designated as non-responsiveness, or exercise resistance (51). This heterogeneity does not arise solely from differences in adherence; rather, it is a complex biological phenomenon deeply rooted in multiple dimensions, encompassing genomic architecture, pathophysiological status, microbial community composition, and the inherent limitations of intervention design itself. The following section systematically deconstructs the multidimensional determinants of exercise resistance, progressing from endogenous individual factors to exogenous intervention-related factors.

### 4.1 Genetic polymorphisms and muscle fiber phenotype: the innate heterogeneous basis of exercise metabolic adaptability

Genetic polymorphisms constitute the foundational determinants of interindividual variation in exercise metabolic adaptability. Among the genetic loci directly implicated in exercise-induced insulin sensitization, functional polymorphisms in PPARGC1A — the gene encoding PGC-1α — can significantly influence the magnitude of mitochondrial biogenesis induction following exercise stimulation. The ACTN3 R577X variant alters the expression level of α-actinin-3 in skeletal muscle and thereby modifies muscle fiber functional characteristics: individuals homozygous for the 577X allele (with complete absence of α-actinin-3) exhibit a greater propensity for metabolic flexibility and aerobic endurance adaptation, whereas those homozygous for the R allele display a more pronounced type II muscle fiber explosive power phenotype — two genotypes that differ fundamentally in their metabolic adaptation patterns in response to distinct exercise modalities. Polymorphisms in PPARG, which encodes PPARγ, indirectly modulate the lipotoxic burden on skeletal muscle by influencing adipocyte differentiation and lipid metabolism (52). The cumulative effects of these genetic variants may result in a significantly attenuated activation of the AMPK/SIRT1/PGC-1α signaling axis in a subset of patients performing equivalent exercise training loads compared with the population mean, with the induction of mitochondrial biogenesis subject to constitutive genetic constraint — thereby establishing, at the genomic level, a predisposition toward low responsiveness to exercise intervention.

Interindividual variation in skeletal muscle fiber type composition further modulates the metabolic benefits conferred by exercise. Mitochondria-rich type I muscle fibers (slow-twitch fibers) are more responsive to aerobic exercise-induced AMPK activation and GLUT4 upregulation, whereas skeletal muscle with a predominance of type II fibers is more adaptable to resistance training, which improves glucose uptake primarily through mTOR/Akt signaling-driven promotion of muscle protein synthesis and increased GLUT4 density (53). It should be noted that excessive mTORC1 activation can phosphorylate IRS-1 at Ser636/639 via ribosomal protein S6 kinase 1 (S6K1), producing an insulin resistance effect analogous to that mediated by JNK/IKKβ — a phenomenon termed the “mTOR paradox” — indicating that the influence of resistance training on insulin signaling is not unidirectionally facilitative, and that precise calibration of training dosage and intensity carries important clinical implications. Accordingly, aerobic exercise and resistance training differ fundamentally in their molecular pathways for improving IR, and the adoption of a uniform exercise modality without regard for individual muscle fiber phenotype and genetic background represents an important structural contributor to exercise resistance in a subset of patients.

### 4.2 Baseline metabolic inflammatory burden: pathological compression of the effective threshold for exercise-induced insulin sensitization

The baseline level of metabolic inflammation in individual patients represents a critical modulatory variable governing the efficacy of exercise intervention, with the core mechanism residing in the marked compression of the tolerance threshold for exercise-induced reactive oxygen species (ROS) imposed by a high baseline inflammatory burden. In patients with T2DM who have been subjected to chronically elevated meta-inflammation, skeletal muscle mitochondria are already in a state of functional impairment due to sustained oxidative damage, with severely depleted compensatory reserves within the endogenous antioxidant buffering system — encompassing the superoxide dismutase (SOD) and glutathione (GSH) systems (54). Against this background, the additional ROS generated by exercise at conventional intensities can rapidly exceed the cellular redox buffering capacity. Compounding this, the NLRP3 inflammasome — already maintained in a primed state by persistent lipotoxic stimulation — is further engaged by the excess exercise-derived ROS, which at this juncture paradoxically function as the second activation signal for NLRP3, promoting the maturation and secretion of IL-1β, exacerbating IKKβ/JNK-mediated serine phosphorylation of IRS-1, and thereby generating a pro-inflammatory countereffect that is diametrically opposed to the intended therapeutic outcome (55).

This mechanism reveals that in patients with a high baseline inflammatory burden, there exists a critical effective intensity window for exercise: below this window, the anti-inflammatory effects are insufficient to counteract the sustained inflammatory driving force; above it, exercise-induced oxidative stress paradoxically activates NLRP3 signaling, further aggravating IR. The width and position of this effective window vary enormously across individuals and are dynamically coupled to the patient’s current inflammatory biomarker profile — including circulating IL-1β and high-sensitivity C-reactive protein (hsCRP) — rendering accurate identification through fixed empirical prescriptions exceedingly difficult. This represents an important mechanistic basis for the inconsistent efficacy of existing standardized exercise protocols in patients with high inflammatory burden.

### 4.3 Gut microbiota dysbiosis and metabolic endotoxemia: systemic attenuation of exercise-induced insulin sensitization

The gut microbiome, as a pivotal regulator of metabolic–immune crosstalk, influences the molecular responsiveness of the organism to exercise stimuli through multiple pathways and represents an important systemic source of clinical heterogeneity. The characteristic dysbiosis observed in T2DM manifests as a reduction in the abundance of short-chain fatty acid (SCFA)-producing bacteria — exemplified by Faecalibacterium prausnitzii — alongside a decline in Akkermansia muciniphila, whose principal function is the maintenance of intestinal mucosal barrier integrity, accompanied by a global impairment of gut mucosal barrier function (56). SCFAs, particularly butyrate, serve not only as the primary energy substrate for colonocytes but also suppress the pro-inflammatory gene expression of immune cells through inhibition of histone deacetylase (HDAC) activity, while directly activating G protein-coupled receptors 41 and 43 (GPR41/43) in peripheral tissues to enhance insulin sensitivity. Dysbiosis-driven reductions in SCFA production consequently abrogate these metaboloprotective effects (57). More critically, compromised intestinal barrier function permits the continuous translocation of lipopolysaccharide (LPS) into the systemic circulation, establishing metabolic endotoxemia. Upon reaching skeletal muscle via the circulation, LPS sustains persistent activation of the myocellular TLR4→IKKβ/JNK signaling cascade, chronically maintaining IRS-1 serine phosphorylation at an elevated baseline, thereby systemically undermining the downstream metabolic sensitization effects induced by AMPK activation (58). Under these conditions, the signaling gain generated by exercise-activated AMPK is concurrently offset — even when the exercise dose itself fully adheres to guideline-recommended standards, the resultant metabolic benefits are substantially attenuated by the persistent background interference of TLR4 signaling. This mechanism indicates that, for specific patient subpopulations, interventions targeting intestinal barrier restoration and microbiome composition optimization — including targeted probiotic or prebiotic supplementation and dietary restructuring centered on increased dietary fiber intake — may represent an indispensable prerequisite for enhancing exercise responsiveness, rather than merely adjunctive measures.

### 4.4 Nonlinear characteristics of the exercise dose–response relationship and the practical gap in precision prescription systems

Beyond the endogenous individual factors discussed above, the inherent design limitations of existing exercise intervention strategies constitute an equally important structural contributor to clinical heterogeneity. Current clinical exercise intervention research predominantly employs fixed moderate-intensity continuous training (MICT) or high-intensity interval training (HIIT) protocols; however, the dose–response relationship of these modalities exhibits pronounced nonlinear characteristics at the individual level. Following weeks to months of sustained stimulation at a given exercise load, the basal activity of AMPK in skeletal muscle is progressively reset, mitochondrial content approaches a new dynamic equilibrium, and the incremental benefits of mitochondrial biogenesis consequently diminish — a phenomenon termed the metabolic adaptation plateau (59). If the intervention protocol lacks a dynamic parameter adjustment mechanism informed by real-time individual metabolic feedback — such as periodic progressive increases in exercise intensity, alternating exercise modalities, or load optimization guided by continuous glucose monitoring — this plateau will rapidly precipitate a stagnation of metabolic sensitization effects, subsequently driving reductions in patient adherence and progressive attenuation of intervention efficacy.

The prevailing mainstream clinical exercise guidelines — including the exercise recommendations issued by the American Diabetes Association (ADA) — are formulated on the basis of population-averaged effects derived from large-scale randomized controlled trials, and therefore cannot adequately capture the high degree of interindividual dispersion in genetic background, baseline inflammatory status, muscle fiber phenotype, and gut microbiome composition. The systematic gap between theoretically projected benefits and actual clinical gains underscores a critical practical deficit in the field of exercise medicine: the absence of a closed-loop precision management system capable of real-time sensing of individual metabolic responses and dynamic optimization of prescription parameters.

In summary, exercise resistance and clinical heterogeneity represent a composite biological phenomenon arising from the multidimensional superimposition of genetic polymorphisms, baseline pathophysiological status, gut microbial community variability, and intervention design limitations. Their resolution cannot rely on the localized optimization of a single biomarker or fixed exercise parameter, but must instead leverage multi-omics technologies to systematically characterize the individual metabolic–immune phenotype, and on this basis, construct a dynamic, individualized precision exercise intervention system. This conceptual framework will be further elaborated in the following section, where the integrative application of multi-omics and the construction of a precision exercise medicine paradigm will be explored.

## 5 Clinical application, controversies, and future perspectives: constructing a closed-loop precision exercise medicine system

The core clinical value of exercise as an intervention in the comprehensive management of T2DM lies in its pleiotropic effects — the multi-pathway synergistic actions that simultaneously target metabolic sensitization, anti-inflammatory remodeling, and mitochondrial quality restoration — a systemic benefit that no single pharmacological agent can replicate. Nevertheless, the translation of the molecular mechanisms described above into precision clinical prescriptions continues to face a number of unresolved key controversies and translational bottlenecks. This section focuses on the central contentious issues within current clinical evidence, a critical appraisal of multimodal combined intervention strategies, and the future construction pathway for a precision exercise medicine system — three dimensions that together constitute a complete discussion framework bridging mechanistic understanding and clinical application.

### 5.1 Core contentious issues in current clinical evidence

#### 5.1.1 Dose–response relationship between exercise intensity and anti-inflammatory efficacy

Although the acute effects of high-intensity interval training (HIIT) in activating AMPK and inducing myokine release have been confirmed across multiple short-term studies, and existing meta-analyses have preliminarily suggested that HIIT achieves anti-inflammatory and insulin-sensitizing effects comparable to, or marginally superior to, those of moderate-intensity continuous training (MICT) (60), the robustness of these conclusions remains disputed due to the heterogeneity of included study protocols and the limited follow-up durations inherent to these analyses. More critically, whether HIIT systematically outperforms MICT in suppressing chronic low-grade inflammation — and particularly in attenuating sustained NLRP3 inflammasome activation — remains insufficiently supported by long-term randomized controlled trial evidence; the majority of comparative studies to date are based on animal models or small-sample short-term human studies, and their conclusions should not be broadly extrapolated.

Concurrently, the clinical question of whether combined aerobic and resistance training protocols are superior to either modality alone remains unresolved. The molecular complementarity of these two exercise types — with aerobic exercise predominantly engaging the AMPK/PGC-1α axis and resistance training primarily activating the mTOR/Akt axis and increasing GLUT4 density (with dosage calibration requiring careful attention to the IRS-1 negative feedback risk posed by excessive mTORC1 activation) — provides a robust theoretical rationale for combined protocols (61). However, at the clinical implementation level, the optimal ratio and sequencing of these modalities to produce maximal synergistic anti-inflammatory and insulin-sensitizing effects awaits clarification through large-scale, long-duration, head-to-head comparative trials.

#### 5.1.2 Identification of exercise responsiveness biomarkers and their clinical translation

The identification of biomarkers capable of reliably predicting individual exercise responsiveness is a prerequisite for precision exercise prescription, and represents one of the most scientifically contested questions in the field. Candidate biomarkers span multiple levels: circulating myokine profiles — including baseline IL-6 levels and the magnitude of exercise-induced dynamic changes, with chronic baseline levels and acute exercise-induced fluctuations being fundamentally distinct in their clinical significance and requiring careful differentiation — skeletal muscle PPARGC1A expression, circulating mitochondrial DNA (mtDNA) as a surrogate index of mitochondrial damage severity, and baseline inflammatory markers including high-sensitivity C-reactive protein (hsCRP) and IL-1β. Irisin has also been proposed as a candidate marker; however, given the substantial methodological variability in its measured baseline concentrations and exercise-induced changes across detection platforms, its validity as a reliable biomarker requires support from standardized assay platforms before clinical adoption (62).

No unified standards currently exist for the detection sensitivity, specificity, or clinical operability of the above candidate biomarkers; furthermore, the sampling time points relative to exercise bouts exert a significant influence on results, and no single biomarker measured in isolation is sufficient to accurately predict an individual’s exercise response pattern. Establishing a multi-biomarker panel for exercise responsiveness — validated through large-scale prospective studies — represents the critical translational node connecting mechanistic research and precision prescription development.

### 5.2 Multimodal combined intervention strategies: theoretical synergy and translational reality

#### 5.2.1 Synergistic intervention of exercise and pharmacotherapy

The synergistic effects of exercise combined with existing antidiabetic agents represent an important direction in current clinical research. Taking GLP-1 receptor agonists as an exemplar, their molecular pathways for improving metabolism — through suppression of glucagon secretion, delayed gastric emptying, and direct activation of skeletal muscle GLP-1 receptors — are complementary rather than overlapping with the AMPK pathway activated by exercise; their combination theoretically enables synergistic repair at multiple nodes of the insulin signaling network, while the anti-inflammatory effects of GLP-1 receptor agonists (through NF-κB activation suppression) may further lower the oxidative stress threshold for exercise, thereby broadening the effective intensity window for exercise in patients with high baseline inflammation (63). However, this synergistic effect currently remains largely at the level of mechanistic inference; the optimal dosage combinations and temporal sequencing for such combination approaches lack sufficient support from high-quality clinical trial data, and the quantification of actual synergistic benefits requires systematic evaluation.

In addition, targeted NLRP3 inflammasome inhibitors have demonstrated potential for synergistic improvement of metabolic outcomes with exercise training in animal models; however, clinical safety and efficacy data for metabolic disease indications are currently extremely limited, the translational pathway remains unclear, and overly optimistic projections regarding their clinical application prospects are not warranted at this stage.

#### 5.2.2 Integrated strategies combining exercise with gut microbiome intervention

In light of the systemic attenuation of exercise responsiveness by gut microbiota dysbiosis described in Section 3, the synergistic effects of targeted gut microbiome interventions — centered on probiotics (exemplified by Akkermansia muciniphila and Lactobacillus species) and prebiotics (inulin, fructooligosaccharides) — combined with exercise have emerged as a field of growing research interest. Preliminary evidence suggests that probiotic supplementation can systemically reduce the basal activation intensity of skeletal muscle TLR4 signaling by restoring intestinal barrier integrity and lowering circulating LPS levels, thereby providing a more favorable molecular microenvironment for exercise-induced AMPK signaling gains (64).

It should be noted that exercise itself can reciprocally influence the composition and diversity of the gut microbiome through multiple mechanisms, including enhanced intestinal blood perfusion, modulation of intestinal motility, and alterations in bile acid cycling, establishing a bidirectional regulatory relationship between the two. This mutually causal interaction pattern renders the determination of causal directionality and dose–effect relationships among microbiome intervention, exercise, and metabolic improvement a core methodological challenge in causal inference within this field — one that awaits resolution through large-scale prospective studies employing multi-omics designs.

#### 5.2.3 Exercise mimetics: a potential alternative pathway for severely compromised patients

For patients with severe T2DM whose cardiopulmonary limitations preclude the completion of effective exercise doses, compounds that directly activate key nodes within exercise signaling pathways — exercise mimetics — offer a theoretically grounded alternative approach. Candidate compounds currently at the basic research stage include direct AMPK activators (such as AICAR) and PPARδ agonists, which have been shown in animal models to activate a subset of the metabolic effects of exercise without actual physical exertion (65).

It must be explicitly stated, however, that no exercise mimetic has yet received clinical approval; certain early candidate compounds — including the PPARδ agonist GW501516 — have been terminated from development following identification of potential carcinogenic risks in animal studies. More fundamentally, the biological benefits of exercise are a systemic result of the synergistic integration of multiple pathways across multiple organs; the targeted activation of a specific signaling node by any single compound cannot fully replicate the full-spectrum metabolic–immune benefits of exercise. Exercise mimetic research should therefore currently be positioned as a basic science exploratory endeavor, and its clinical translational potential should be assessed with appropriate caution pending the accumulation of adequate safety and efficacy evidence.

### 5.3 Future construction pathways for precision exercise medicine

It should be emphasized that the ‘precision exercise medicine’ framework proposed herein remains at the stage of theoretical construction and roadmap planning. The translation of this conceptual model into a functional clinical system will require a multi-stage translational research process: first, the identification and screening of clinically robust biomarkers through multi-omics technologies; second, the prospective validation of these biomarkers in diverse T2DM patient cohorts; and finally, the integration of validated data into artificial intelligence-driven clinical decision support tools. We fully acknowledge that the clinical application of this framework is currently constrained by the absence of standardized assay platforms, the scarcity of real-time monitoring data, and insufficient validation of predictive algorithms—all of which represent critical bottlenecks that future research must urgently address.

The construction of a precision exercise medicine system follows a logical progressive framework of “data foundation → decision-support tools → boundary reflection”: first, the systematic characterization of individual exercise responsiveness phenotypes through multi-omics technologies; subsequently, the development of digital closed-loop prescription management tools on this basis; and finally, the explicit prioritization of future research directions premised on a clear-eyed recognition of current research limitations.

#### 5.3.1 Multi-omics integration and individualized metabolic phenotyping

The data foundation for realizing precision exercise medicine lies in the systematic characterization of the individual “exercise responsiveness phenotype.” Analysis at the level of a single biomarker or a single omics layer is insufficient to capture the multidimensional biological determinants of exercise resistance. Future research must integrate genomics (to identify key polymorphic loci influencing exercise adaptation), transcriptomics (to resolve the dynamic remodeling of skeletal muscle gene expression networks before and after exercise), metabolomics (to track exercise-induced changes in circulating metabolite profiles), and gut metagenomics (to characterize interindividual differences in microbiome composition and function), thereby constructing multi-omics-driven predictive models of exercise responsiveness. The ultimate form of such a model is an individualized decision-support tool capable of stratifying patients by “metabolic–immune phenotype” prior to exercise intervention and accordingly matching the optimal combination of exercise type, intensity, and frequency.

Nevertheless, a significant translational gap exists between multi-omics discoveries and clinical application. To build effective clinical decision support tools, a rigorous translational bridge must be established. First, biomarker panels with high sensitivity and specificity must be screened and validated across different T2DM subpopulations through multi-omics integration technologies. Second, these biomarkers must be correlated with real-time physiological monitoring data to characterize their dynamic response profiles during exercise interventions. Finally, before incorporating these biomarkers into computational systems, their predictive performance must undergo external validation through large-scale prospective clinical trials to ensure reliability and safety in real-world clinical settings.

#### 5.3.2 Digital technology-supported closed-loop exercise prescription systems

The integration of multi-omics information with real-time physiological monitoring data represents the critical technical pathway for constructing a closed-loop precision exercise prescription management system. At the level of real-time monitoring, the multimodal data fusion of continuous glucose monitoring (CGM), heart rate variability (HRV), and wearable exercise load sensors can theoretically provide real-time reflection of an individual’s metabolic stress state during exercise, enabling identification of whether the individual is in a phase of effective adaptation or excessive physiological stress. At the decision-support level, artificial intelligence engines based on mechanistic metabolic models and machine learning algorithms can transform these multidimensional physiological data into dynamic prescription adjustment recommendations, with preliminary technical validation demonstrating potential for identifying metabolic adaptation plateaus and signaling the appropriate timing for prescription parameter modifications (66).

It should be noted that this closed-loop system remains at the stage of technical validation and small-scale clinical piloting; its effectiveness, safety, and health economic value in large-scale T2DM management require systematic verification through rigorously designed large-scale randomized controlled trials, and it should not be promoted as a mature clinical tool at this stage.

#### 5.3.3 Research limitations and future directions

In synthesizing the existing evidence in this review, the following structural limitations must be acknowledged.

First, current mechanistic research on exercise intervention is predominantly based on animal models and short-term small-sample human studies; high-quality evidence from long-term (exceeding one year), large-scale randomized controlled trials is comparatively scarce, and the extrapolation of mechanistic conclusions to real-world populations warrants caution. Second, the majority of mechanistic studies have employed healthy volunteers or subjects with simple obesity, and the translational applicability of their conclusions to real-world T2DM patients with multiple comorbidities remains questionable. Third, the degree of standardization of exercise intervention protocols — encompassing intensity, modality, duration, and supervision mode — varies substantially across studies, constraining the cross-study reliability of meta-analytic conclusions. Fourth, female participants and elderly T2DM patients (aged >70 years) have been systematically underrepresented in existing exercise intervention research; yet these two subgroups possess important physiological specificities in terms of estrogen levels, skeletal muscle fiber composition, and baseline inflammatory background, and their exercise-related molecular response patterns cannot be directly extrapolated from general adult male samples. In summary, advancing the ‘precision exercise medicine’ framework from the laboratory to clinical application still faces multiple practical barriers. First, the lack of standardized biomarker assay platforms renders cross-study data comparison difficult. Second, current research is predominantly based on controlled experimental settings, with a critical shortage of long-term, large-scale clinical evidence from real-world T2DM patient populations. Third, existing computational models have yet to integrate multidimensional data encompassing patients’ genetic backgrounds, gut microbiota, and lifestyle factors. Overcoming these barriers requires not only deep integration of exercise physiology with clinical medicine but also the accumulation of high-quality, real-world research data from multicenter studies—an essential prerequisite for realizing a closed-loop precision exercise prescription management system.

Based on the above limitations, priority directions for future research should include: establishing a multi-biomarker panel for exercise responsiveness validated through large-scale prospective multi-omics studies; conducting stratified exercise intervention randomized controlled trials in T2DM subpopulations with high baseline inflammatory burden; systematically pursuing dedicated mechanistic exercise intervention studies in female and elderly T2DM subgroups to address the demographic gaps in the existing evidence base; and systematically evaluating optimal synergistic protocols for the combined application of exercise and novel anti-inflammatory agents, including NLRP3-targeted inhibitors and GLP-1 receptor agonists. The advancement of these research priorities depends upon deep interdisciplinary collaboration among exercise physiology, clinical endocrinology, multi-omics bioinformatics, and digital health engineering.

In summary, the clinical value of exercise in the comprehensive management of T2DM has long surpassed the traditional framework of caloric expenditure; its molecular mechanisms as a systemic metabolic–immune modulator have been progressively elucidated. Nevertheless, the clinical translation from mechanistic understanding to precision prescription still requires surmounting multiple translational bottlenecks — including the resolution of genetic heterogeneity, biomarker validation, multi-omics predictive model construction, and digital closed-loop tool development. Achieving substantive progress in this translational process constitutes the core mission of precision exercise medicine in the field of T2DM for the next stage of research.

## 6 Conclusions

By synthesizing existing molecular findings into a coherent ‘Muscle–Immune–Metabolic’ framework, this review offers a new perspective on T2DM management. In contrast to traditional descriptive reviews, the ‘precision exercise medicine’ framework we propose shifts the focus from isolated molecular targets to a dynamic, multi-omics-driven system, providing a roadmap for future clinical translation.

1. The pathological essence of T2DM is the systemic impairment of insulin signaling pathways by chronic meta-inflammation — encompassing M1 polarization of adipose tissue macrophages, IKKβ/JNK-mediated multi-site serine phosphorylation of IRS-1, oxidative stress-driven amplification of NLRP3 inflammasome activation, and the systemic inflammatory gain perpetuated by mitochondrial dysfunction and gut microbiota dysbiosis. Together, these processes constitute a reversible, multi-node pathological cascade, providing the fundamental biological rationale for physiological intervention.

2. Exercise achieves targeted repair of the above multi-node impairments through AMPK-driven metabolic sensitization, myokine-mediated systemic anti-inflammatory effects, mitophagy-based mitochondrial quality control, and cross-organ metabolic synergy among skeletal muscle, the liver, and adipose tissue. This cross-organ, multi-pathway synergistic repair effect constitutes the fundamental biological advantage of exercise intervention over any single pharmacological agent.

3. The interaction among genetic polymorphisms, baseline inflammatory burden, gut microbiome composition, and exercise protocol design renders exercise responsiveness highly individualized, manifesting as a distinct metabolic–immune phenotype for each patient. Uniform exercise guidelines formulated on the basis of population-averaged effects are therefore inherently unable to address the full-spectrum needs of real-world T2DM patients.

It must be acknowledged that the current conceptual framework remains substantially reliant on animal models, small-sample short-term studies, and highly heterogeneous meta-analyses, with inherent limitations in evidence grade and extrapolation reliability; the systematic underrepresentation of female and elderly subgroups further constrains the generalizability of existing conclusions. Achieving a fundamental translational leap from mechanistic understanding to precision clinical application will require the coordinated advancement of multi-omics-driven exercise responsiveness biomarker systems, individualized stratified intervention clinical trials, and digital closed-loop prescription tools. Only when exercise intervention truly achieves individualized, dynamic, and quantifiable precision management will its full clinical therapeutic potential in the long-term comprehensive management of T2DM be systematically realized.
